# Outcomes of Sepsis and Septic Shock in Cancer Patients: Focus on Lactate

**DOI:** 10.3389/fmed.2021.603275

**Published:** 2021-04-26

**Authors:** René López, Rodrigo Pérez-Araos, Fernanda Baus, Camila Moscoso, Álvaro Salazar, Jerónimo Graf, José Miguel Montes, Suraj Samtani

**Affiliations:** ^1^Departamento de Paciente Crítico, Clínica Alemana de Santiago, Santiago, Chile; ^2^Escuela de Medicina, Facultad de Medicina Clínica Alemana - Universidad del Desarrollo, Santiago, Chile; ^3^Escuela de Kinesiología, Facultad de Medicina Clínica Alemana - Universidad del Desarrollo, Santiago, Chile; ^4^Medical Oncology, Fundación Chilena de Inmuno Oncologia, Santiago, Chile; ^5^Medical Oncology Service, Clinica Bradford Hill, Santiago, Chile

**Keywords:** cancer, intensive care unit, septic shock, oncological patient, cancer prevention and control

## Abstract

The number of oncological patients (OP) admitted to intensive care units (ICU) for sepsis/septic shock has dramatically increased in recent years. The definition of septic shock has been modified, adding hyperlactatemia as a severity biomarker for mortality. However, it remains poorly reported in septic OP. We performed a retrospective analysis from a prospective database of sepsis/septic shock patients admitted to our ICU between September 2017 and September 2019 and followed until day 90. We identified 251 patients and 31.9% had active oncological comorbidity, mainly solid tumor (81.3%). Septic shock criteria were met for 112 (44.6%). Hyperlactatemia was observed in 136 (54.2%) patients and this was associated with a lower survival rate. Overall 90-day mortality was 15.1%. In OP vs. non-OP, hyperlactatemia was more frequent (65% vs. 49.1%, *p* = 0.013) and associated with lower survival (65.4% vs. 85.7%, *p* = 0.046). In OP, poor performance status was also associated with lower survival (HR 7.029 [1.998–24.731], *p* = 0.002) In an adjusted analysis, cancer was associated with lower 90-day survival (HR 2.690 [1.402–5.160], *p* = 0.003). In conclusion, septic OP remains a high mortality risk group in whom lactate levels and performance status could help with better risk stratification.

## Introduction

Relevant advances in diagnosis and treatment of oncological patients (OP) have been reported in the past few years, with a significant improvement in their survival rates (1). Additional to cancer therapy advances, improvements in intensive care unit (ICU) support and admission policies have also have contributed to improving survival outcomes (2). The need for objectivity (3) has led to research about specific care for critically ill cancer patients (4–9) achieving better outcomes. Oncological patients account for up to 20% of ICU admission and sepsis denotes a leading reason for ICU admission in this group of patients (10). A higher prevalence of sepsis has been reported in OP vs. non-oncological patients (non-OP) (11). Immunosuppression due to underlying malignancy or its treatment can increase the risk for severe infections (12). Therefore, cancer patients are recognized as a high-risk group for sepsis with high mortality (13). However, in recent decades, better short-term outcomes have been reported in OP admitted to ICU, even in the subgroup of patients with a need for vasopressor support (1). On the other hand, sepsis is one of the leading causes of death and critical illness in the world (14). Sepsis is a life-threatening organ dysfunction as a result of infection and dysregulated host response (15). When it is associated with cellular dysfunction (evidenced as hyperlactatemia) and the need for vasopressor despite appropriate fluid reanimation, septic shock is established and its mortality is close to 40% (16).

The most recent consensus on the definition of septic shock emphasizes higher mortality rates when vasopressor is needed and hyperlactatemia is present (16). However, prognostic markers are usually inferred from non-OP and might not as accurate in OP admitted to ICU. As an example, central venous saturation has been classically associated with worst outcomes; however, in a recent trial, it was not associated with an early complication in cancer patients presenting in the emergency department (17).

Data related to lactate in septic cancer patients is lacking. Moreover, studies regarding lactate levels or hyperlactatemia in septic cancer patients are underreported (1, 18). Therefore, this study aimed to describe survival rates in OP and non-OP patients according to hyperlactatemia status.

## Materials and Methods

### Study Design and Patients

We performed a retrospective analysis from a prospective database as part of project “Registro prospectivo de pacientes ingresados a unidad de cuidados intensivos (RUCI)” in Clínica Alemana de Santiago, a university teaching hospital. All patients admitted between September 24, 2017, and September 21, 2019, were considered. They were followed until day 90 from ICU admission and mortality outcome was recorded. For patients with more than one ICU admission in this period, only the first was taken into account. This project was approved under protocol number 53-2012 by local ethical board “comité científico—ético of Clínica Alemana de Santiago” (IRB00011516), addressed in Av. Vitacura 5951, Santiago of Chile. Informed consent was obtained from each patient or relatives.

### Variables of Interest and Definitions

- *Oncological patients:* Those who have a histological diagnosis of neoplasm and lower than 5 years of remission. (19).
- *Performance status:* We used the Eastern Cooperative Oncology Group (ECOG) score (20).
- *Severity at ICU admission:* We used the Acute Physiology and Chronic Health Evaluation II (APACHE II) score.
- *Sepsis related organ dysfunction:* We used Sequential Organ Failure Assessment (SOFA) score.
- *Sepsis:* Defined as proven or suspected infection with organ dysfunction associated, in agreement with Sepsis-3 consensus definition (15).
- *Sepsis related hyperlactatemia:* Arterial lactate level equal or > 2 mmol/L in a septic patient at ICU admission.
- *Sepsis-3 definition of septic shock:* In agreement with the last consensus definition, patients with proved or suspected infection and need for vasopressor support to achieve a mean arterial pressure of 65 mmHg and hyperlactatemia higher or equal to 2 mmol/L were categorized as septic shock (15).
- *Sepsis-2 definition of septic shock:* Patients with proved or suspected infection and need for vasopressor support to achieve a mean arterial pressure of 65 mmHg despite appropriate fluid therapy (21).
- *Vasopressor treatment:* Patients treated with noradrenaline to achieve a mean arterial pressure at least of 65 mmHg after appropriate fluid therapy.
- *Outcome:* Survival at day 90.

### Statistical Analysis

First, we did a descriptive analysis of the whole group and then a characterization according to oncological status. Oncological patients was also described according to neoplasm type and performance status. Quantitative variables were described as mean (SD) and were compared between groups using an unpaired *t*-test. Distributions were explored by the *Kolmogorov-Smirnov* test. In agreement with the central limit theorem, the sample size allowed for the appropriate use of a parametric test with better rigor than a non-parametric test independently of sample distribution (22, 23). In the same way, in accordance with *Skovlund* and *Fenstad* (22), our sample meets the conditions for parametric test use. Qualitative variables were described as frequency (percentages) and were compared between groups by Fisher's exact test. The outcome was assessed using survival analysis and an adjusted comparison between OP and non-OP patients' survival was performed by Cox regression. To compare survival curves between patients with or without hyperlactatemia, we used the Log-Rank test. Significance was defined as *p* < 0.05. Statistical analysis was performed using the SPSS software, version 20.0 (SPSS, Chicago, IL, USA).

## Results

We identified 251 patients who meet sepsis-3 criteria for sepsis or septic shock. Patients were mainly male (57%) and they were 64.7 (18.4) years old. A moderate severity with an APACHE II score of 15.6 (7.9) points was observed. The source of infection was mainly pulmonary or digestive. 32.3% were admitted in the postoperative setting. A 44.6% meet septic shock criteria in agreement with the Sepsis-3 task force while 69.7% needed vasopressor. Hyperlactatemia > 2 mmol/L was seen in 54.2%. Hundred and forty-five patients were supported with invasive mechanical ventilation (IMV), however, only 46 of them meet Berlin's criteria for acute respiratory distress syndrome. Overall, the 90-day mortality rate was 15.1%. A detailed patients description is shown in Table 1.

**Table 1:** Patients' characterization according to oncological or non-oncological status.

**Variable**	**All**	**OP**	**non-OP**	***P*-value**
	***N* = 251**	***N* = 80**	***N* = 171**	
**DEMOGRAPHICS**				
Age, years	64.7 (18.4)	67.7 (11.9)	63.4 (20.7)	0.039
Male, *N* (%)	143 (57.0)	51 (63.8)	92 (53.8)	0.089
APACHE II, points	15.6 (7.9)	17.8 (6.8)	14.7 (8.2)	0.004
SOFA, points	6.8 (3.5)	7.1 (3.5)	6.7 (3.4)	0.377
AKI at admission, *N* (%)	106 (42.2)	36 (45.0)	70 (40.9)	0.318
ARDS at admission, *N* (%)	46 (18.3)	15 (18.8)	31 (18.1)	0.517
IMV, *N* (%)	145 (57.8)	50 (62.5)	95 (55.6)	0.184
Surgical, *N* (%)	81 (32.3)	29 (36.3)	52 (30.4)	0.218
Lactate, mmol/L	2.9 (2.9)	2.9 (2.0)	2.9 (3.3)	0.938
Hyperlactatemia, *N* (%)	136 (54.2)	52 (65.0)	84 (49.1)	0.013
Septic shock, *N* (%)	112 (44.6)	42 (52.5)	70 (40.9)	0.057
**SOURCE**				0.082
Bacteremia, *N* (%)	7 (2.9)	1 (1.3)	6 (3.5)	
Pulmonary, *N* (%)	80 (31.9)	21 (26.3)	59 (34.5)	
Digestive, (%)	94 (37.5)	40 (50.0)	54 (31.6)	
Urinary, *N* (%)	32 (12.7)	6 (7.5)	26 (15.2)	
Skin and soft tissue, *N* (%)	11 (4.4)	3 (3.8)	8 (4.7)	
Other, *N* (%)	27 (10.8)	9 (11.3)	18 (10.5)	
**OUTCOMES**				
ICU LOS, days	8 (9)	9 (10)	7 (9)	0.353
90-day mortality, *N* (%)	38 (15.1)	22 (27.5)	16 (9.4)	<0.001

*OP, oncological patient; non-OP, non-oncological patient; APACHE II, acute physiology and chronic health evaluation II score; SOFA, sequential organ failure assessment score; AKI, acute kidney injury at admission; ARDS, acute respiratory distress syndrome; IMV, invasive mechanical ventilation; ICU LOS, intensive care unit length of stay*.

We identified 80 (31.8%) as OP; being 81.3% solid tumor and 65.7% were stage IV. Interestingly, 70.1% were ECOG 1 (Table 2). In comparison with non-OP, cancer patients were elderly and had more severe illnesses. Hyperlactatemia higher than 2 mmol/L was more frequent in OP (65% vs. 49.1%, respectively; *p* = 0.013).

**Table 2:** Patients' characterization according to oncological or non-oncological status.

**Oncological characteristics**	**Patients, *N* (%)**
Hematological	15 (18.7)
Solid	65 (81.3)
Lung	6 (9.2)
Breast	5 (7.7)
Colon	9 (13.8)
Gastric	3 (4.6)
Other	41 (63.1)
**Stage**	
I	2 (2.9)
II	9 (12.9)
III	13 (18.6)
IV	46 (65.7)
**ECOG**	
1	54 (70.1)
2	18 (23.4)
3	5 (6.5)

*ECOG, East cooperative oncology group performance status*.

In OP, a significant association with lower survival was observed when the results were categorized according to hyperlactatemia (yes 65.4% vs. no 85.7%, *p* = 0.046), but not when they were categorized according to vasopressor need (yes 69.5% vs. no 81%, *p* = 0.336).

**Figure 1:**
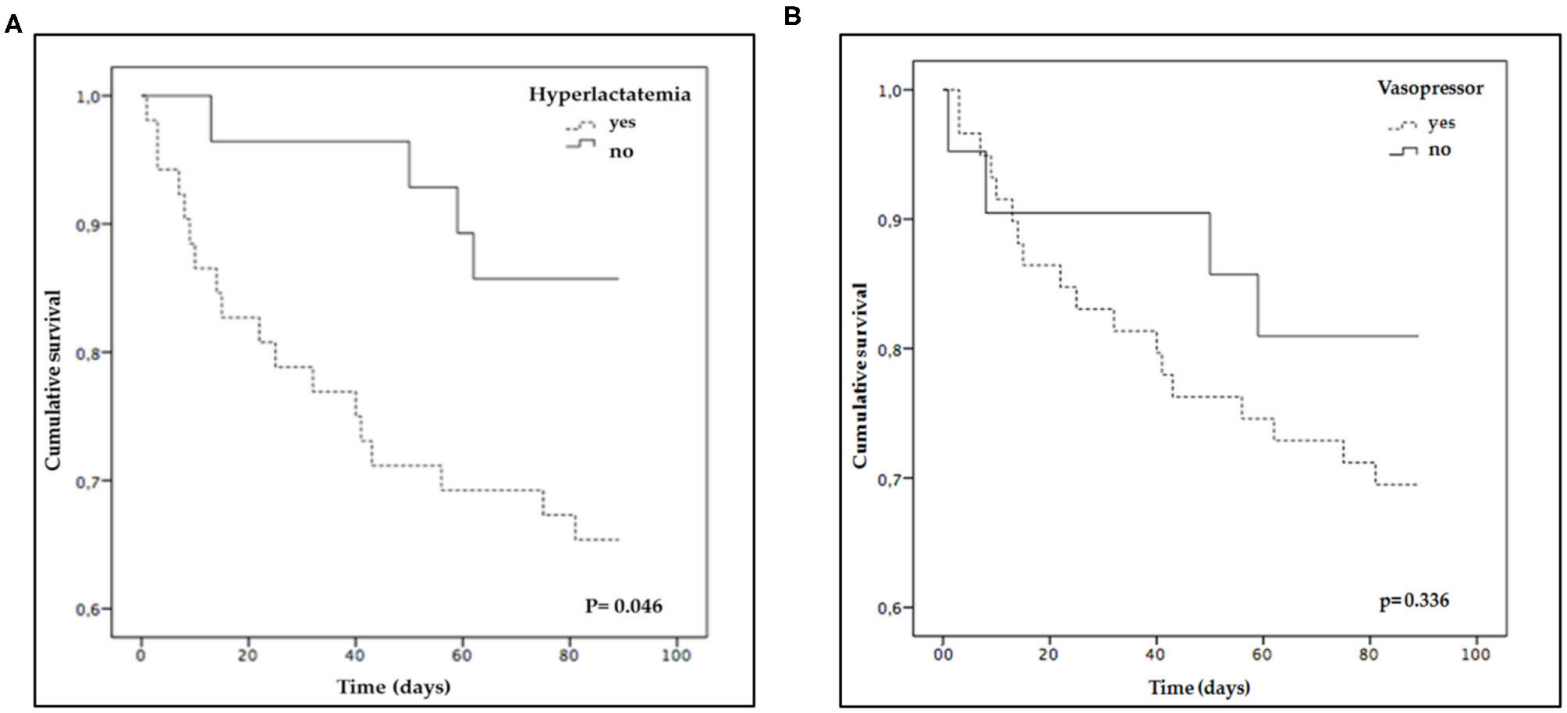
Survival curves of oncological patients. The curves comparison was performed using Log-Rank test: **(A)** Categorized according to hyperlactatemia (>2 mmol/L); **(B)** Categorized according to vasopressor need.

**Figure 2:**
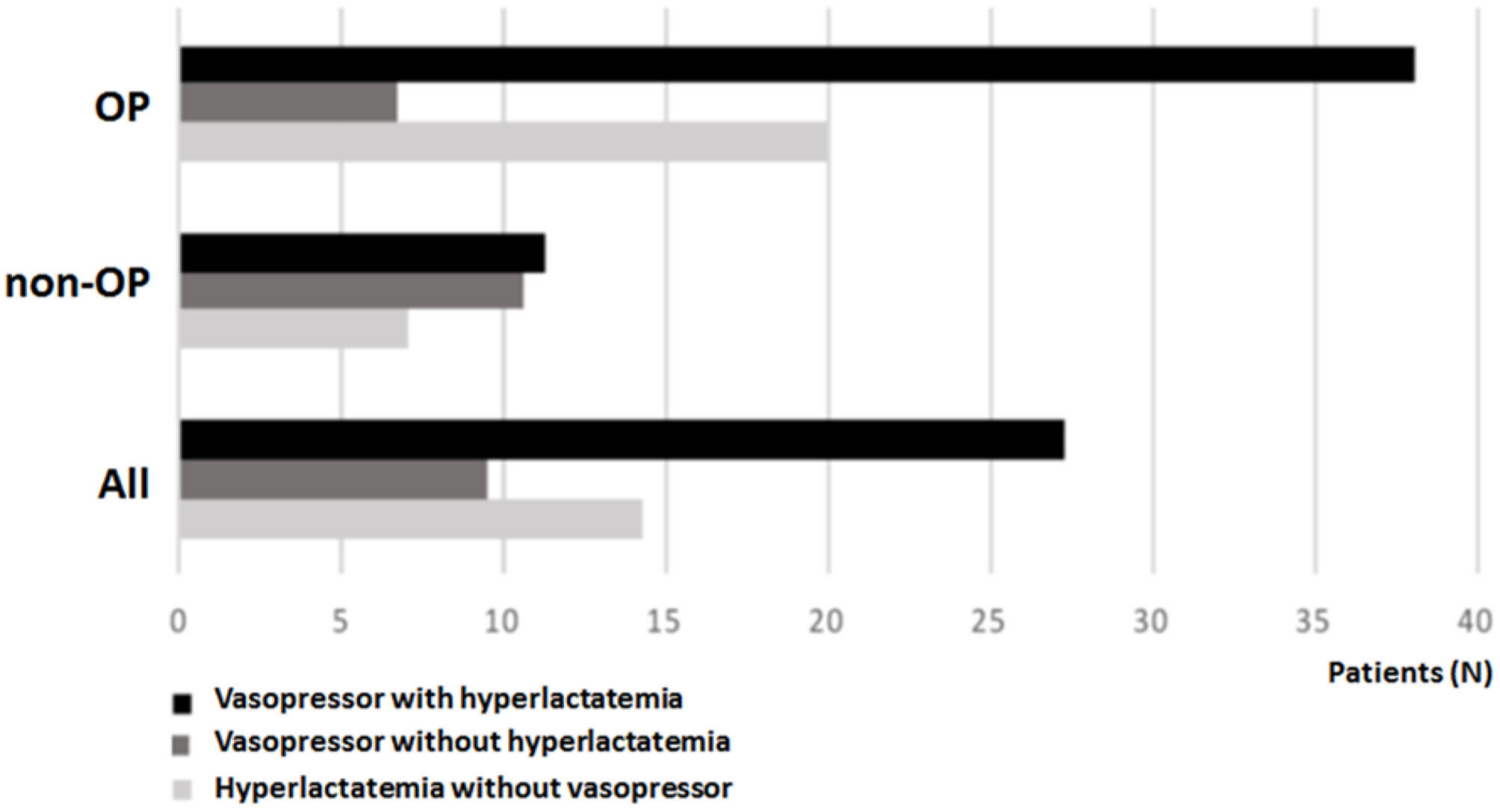
Outcome distribution in oncological, non-oncological and all patients according to vasopressor need with hyperlactatemia (>2 mmol/L), vasopressor need without hyperlactatemia, and hyperlactatemia (>2 mmol/L) without vasopressor need.

The overall 90-day mortality rate was higher in OP vs. non-OP (27.5% vs. 9.4%, respectively, *p* < 0.001). The distribution of outcomes according to hyperlactatemia and vasopressor need between OP and non-OP are shown in Figures 1, 2.

**Figure 3:**
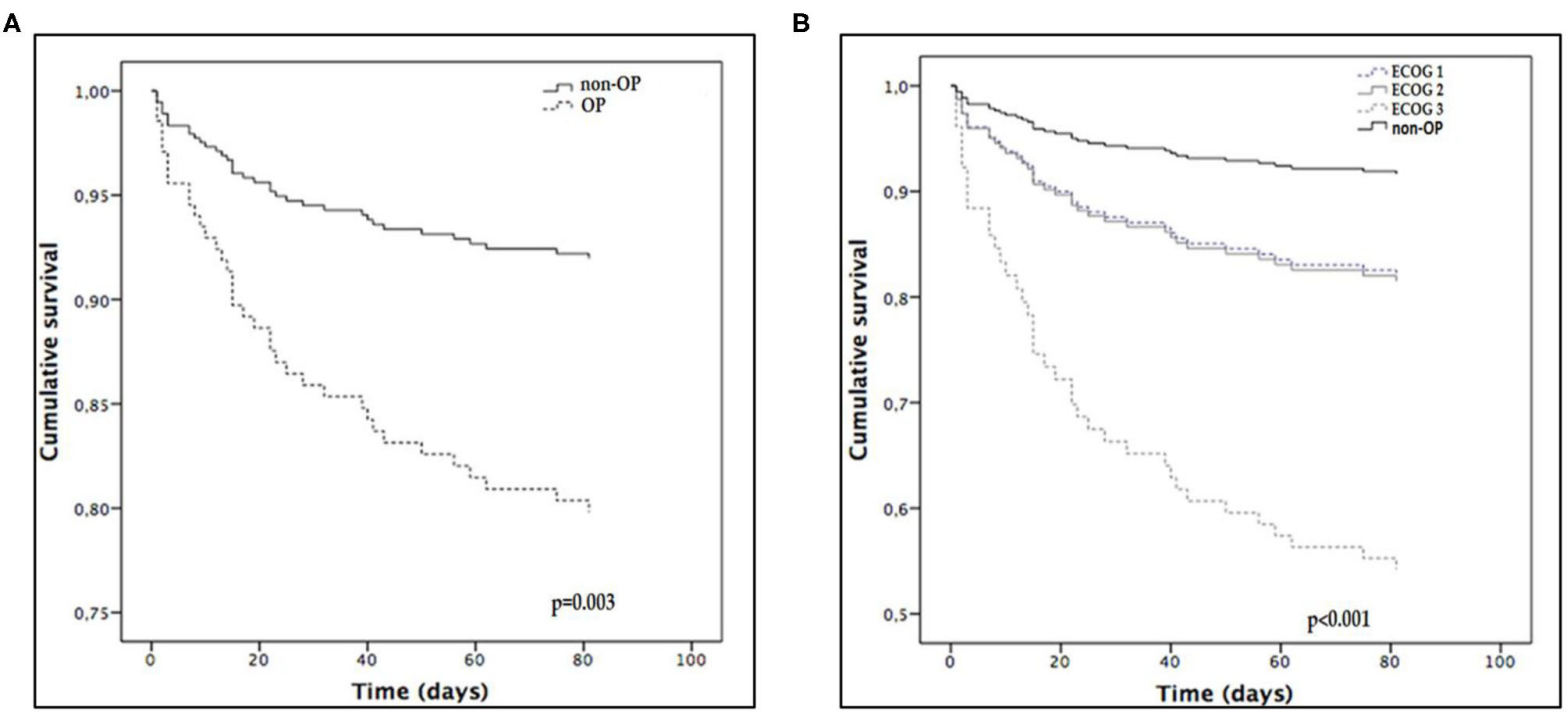
Adjusted survival curves where OP is oncological patients. **(A)** non-OP vs. OP; **(B)** non-OP (as reference) and OP categorized according to ECOG performance status. ECOG, East cooperative oncology group.

In a survival analysis adjusted by APACHE II score, SOFA score, hyperlactatemia, and surgical admission, we found a lower survival in OP with a hazard ratio (HR) of 2.690 [1.402–5.160], *p* = 0.003 (Figure 3A). When outcome performance was assessed according to ECOG status, patients with ECOG 1-2 had lower survival than non-OP but better survival rates than patients who were ECOG-3 (Figure 3B).

**Figure 4:**
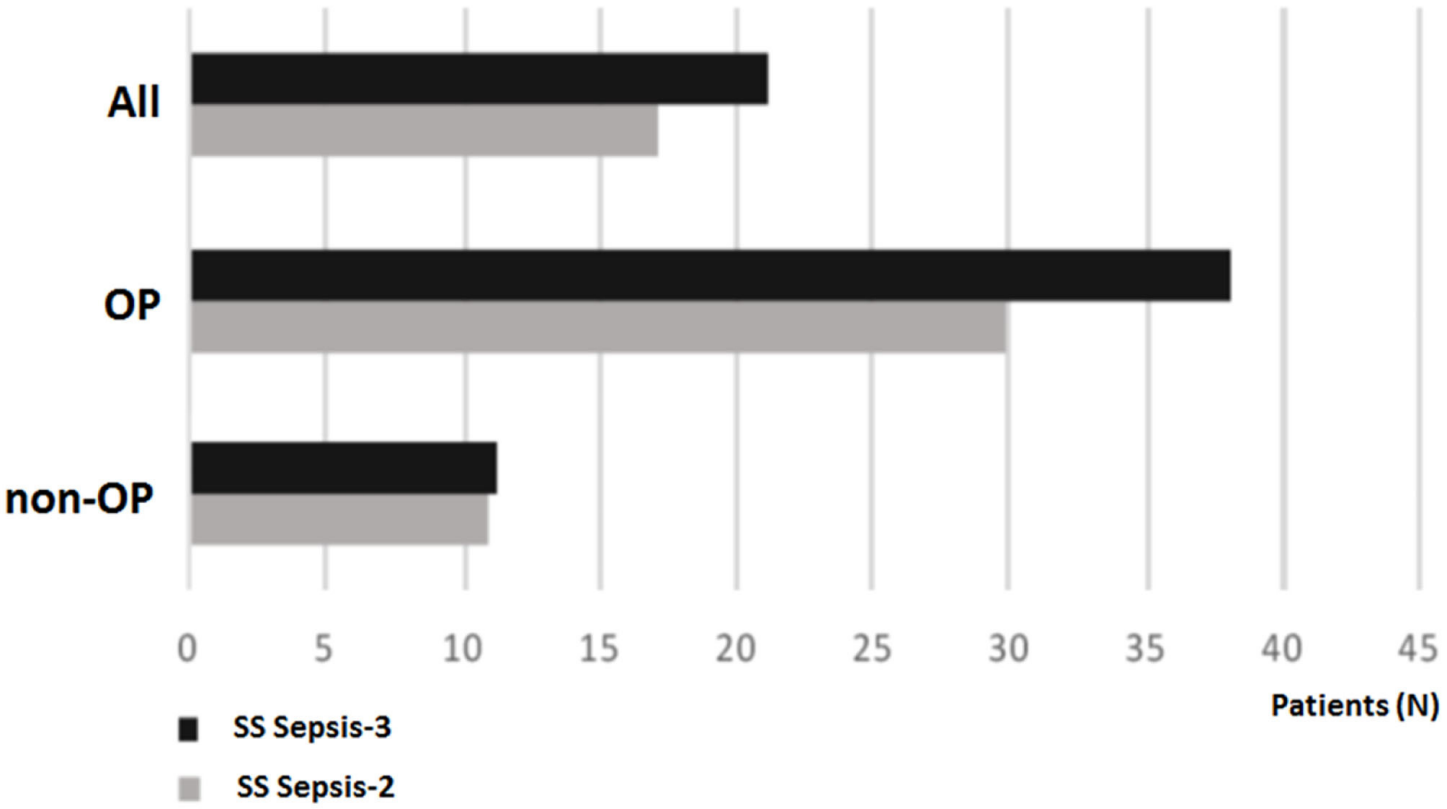
Outcome in all patients, oncological patients (OP) and non-OP, according to septic shock task force definition. SS Sepsis-3, Septic shock according to Sepsis-3 task force; SS Sepsis-2, Septic shock according to Sepsis-2 task force.

Finally, only the outcomes for OP were sensitive to septic shock definition (Sepsis task force 2 or 3, Figure 4).

## Discussion

The main finding of this study was a lower survival rate in OP vs. non-OP with sepsis/septic shock. The antecedent of neoplasm was an independent variable associated with worse outcomes. Remarkably, in our clinical institution, close to a third of patients admitted by sepsis/septic shock were OPs. Among OP, those with poor performance status were independently associated with worse survival. Interestingly, in our patients, hyperlactatemia was associated with lower survival mainly in OP. Likewise, mortality in OP was sensitive to septic shock definition, while in non-OP a relatively low mortality rate with both definitions was observed.

We also observed an overall unadjusted mortality in the lower limit to that reported in other literature and in agreement with a previous report (15). Patients with the sepsis-3 definition of septic shock were a high mortality risk group. Classically, OPs are considered as a group of high risk for infection and present higher mortality rates (13). However, significant improvements in outcomes in these patients have been recently reported (18). Mortality rates over 50% in OP were reported during the 1990s while more recently, cancer patients with sepsis/septic shock mortality rates lower than 35% have been reported (18). Therefore, our results are in agreement with the hypothesis that cancer patients with sepsis are a high-risk group in terms of worse outcomes, but these results also indicate improvement in outcomes in this group in the past few years

Lactate is a biomarker classically linked with worse outcomes in sepsis (18, 19, 22–24) but an elevated serum lactate level is not specific for cellular dysfunction in sepsis (24–27). Specifically, during the course of an infection, an increased lactate >2 mmol/L has been consistently associated with increased mortality (16, 23–25). Moreover, in the last septic shock consensus definition, hyperlactatemia is part of diagnosis criteria (15). Adding hyperlactatemia to continuous vasopressor therapy achieves a better selection of patients with worse outcomes (15, 16). However, in previous studies regarding outcomes in septic cancer patients, lactate levels or frequency of hyperlactatemia are underreported (1, 18).

In our study, OP was recognized as a sensitive group to septic shock outcome according to the definition used. Similarly, Costa et al. found a higher mortality rate in cancer patients with septic shock according to sepsis-3 definitions in comparison with sepsis 2 definitions (27, 28). We found different survival curve behaviors according to whether hyperlactatemia was present or not in septic cancer patients at admission. This was an expected but not obvious finding. For example, venous central saturation, another classical parameter with prognostic value in cancer patients, had not been associated with worse outcomes (17). However, our findings demonstrate that lactate could be a valuable tool in septic cancer patient evaluation. Therefore, lactate levels should be assessed in all patients with suspected sepsis and especially in OP.

This approach is currently being taken into consideration in our clinical practice. In our center, all patients with proven or suspected infection are stratified using lactate levels and if this biomarker is equal or higher than 2 mmol/L, patients are admitted to critical care (intensive care unit or intermediate care unit according to organ dysfunction at admission). In the same way, a clinical researcher should be alert to lactate assessment at admission and include it in future reports regarding outcomes in cancer patients with sepsis.

Another remarkable result was the differentiation of survival according to performance status. This finding is in agreement with data published last year, where intensive care support was followed by better outcomes in cancer patients with good performance status (20, 21, 29). The improvement in outcomes in the last year of critically ill cancer patients and a better patients' risk stratification should lead to the actualization of ICU admission policies (30, 31).

Our study has some limitations and our findings should be taken carefully. First, this is a retrospective analysis; however, the database was prospectively collected. Second, this is a single center study with a relatively small sample, and external validity is limited. Likewise, due to sample size limitations, specific cancer patients' subgroups such as neutropenic or hematological information were not independently analyzed. However, this study achieved the important finding in terms of its reappraisal of lactate in cancer patients with sepsis and invites others to take into account this biomarker in clinical and research settings. The strengths of the study include that this data indicates different survival rates according to oncological status, septic shock definition, and hyperlactatemia status. Moreover, our patients were followed up for 90 days while most studies on sepsis outcomes in cancer patients take into account a follow-up of 30 days or are limited to hospital stay. We also provide a comparison with non-OP patients.

## Conclusions

The outcome in ICU OP with sepsis has improved in recent years, however, these patients remain a high mortality risk group, especially those with poor performance status. Lactate should be used as a biomarker for risk stratification in cancer patients with suspected sepsis. Outcome improvement and better patient stratification could lead to the actualization of ICU admission policies.

## Data Availability Statement

The original contributions presented in the study are included in the article/supplementary material, further inquiries can be directed to the corresponding author/s.

## Ethics Statement

The studies involving human participants were reviewed and approved by Comite de Etica Clinica Alemana. The patients/participants provided their written informed consent to participate in this study.

## Author Contributions

RL and SS contributed to conceptualization, methodology, and study design. RP-A, RL, and SS are responsible for data management. RL, RP-A, JM, JG, and SS. undertook formal analysis, writing, and original draft preparation. All authors contributed to the article and approved the submitted version.

## Conflict of Interest

The authors declare that the research was conducted in the absence of any commercial or financial relationships that could be construed as a potential conflict of interest.

## Abbreviations

OP: oncological patients
ICU: intensive care unit
non-OP: non-oncological patients
ECOG: Eastern cooperative oncology group
APACHE II: acute physiology and chronic health evaluation II
SOFA: sequential organ failure assessment
IMV: invasive mechanical ventilation
ARDS: acute respiratory distress syndrome
ICU LOS: intensive care unit length of stay.
